# Husbands’ concerns and experiences with the progesterone vaginal ring in three sub-Saharan African countries: a mixed methods study

**DOI:** 10.1080/26410397.2022.2104680

**Published:** 2022-08-17

**Authors:** Francis Obare, Fatou Mbow, Saumya RamaRao, Avishek Hazra

**Affiliations:** a Associate, Population Council, Avenue 5, Rose Avenue, P.O. Box 17643, Nairobi 00500, Kenya.; b Country Representative, Population Council, Dakar, Senegal; c Senior Associate, Population Council, New York, NY, USA; d Senior Program Officer, Population Council, New Delhi, India

**Keywords:** self-care interventions, progesterone vaginal ring, men’s influence, women’s rights and autonomy, sub-Saharan Africa

## Abstract

The introduction of progesterone vaginal ring (PVR) in sub-Saharan Africa calls for insights on the product’s role in promoting women’s autonomy regarding their reproductive decision-making and behaviour. Such insights could inform the positioning of the method within family planning programmes in the region. In this paper, we explore husbands’ experiences with PVR as perceived by their wives and as reported by husbands of a subset of women users in Kenya, Nigeria, and Senegal. We discuss how such experiences might influence women’s rights and autonomy regarding their reproductive decisions and contraceptive behaviour. We use a mixed-methods approach drawing on data from quantitative interviews with 174 women and qualitative in-depth interviews with 10 husbands of a subset of the women in the three countries. The findings show that husbands appreciated PVR’s attributes relating to user-control (women could insert and remove the method themselves), ease of use, and non-interference with sex and flow of breast milk. Wives’ perceptions of their husbands’ experiences regarding PVR’s non-interference with sex were consistent with the husbands’ own reports. In addition, health care providers played important roles in supporting sustainable use of the method through giving information, counselling, and assisting women who experienced ring slippage to manage those challenges. The findings suggest that self-managed health technologies such as PVR could expand women’s choices and control over their reproductive decisions. The findings further suggest that sustainable use of such products could require linkages with appropriate health systems structures to address challenges with use if and when they arise.

## Résumé

L’introduction de l’anneau vaginal à la progestérone (AVP) en Afrique subsaharienne exige des éclaircissements sur le rôle du produit dans la promotion de l’autonomie des femmes concernant leur prise de décision et leur comportement en matière de procréation. Ces éclaircissements pourraient guider le positionnement de la méthode dans les programmes de planification familiale de la région. Dans cet article, nous étudions l’usage de l’AVP par les maris tel que le perçoivent leurs épouses et tel qu’il est rapporté par les conjoints d’un sous-ensemble d’utilisatrices au Kenya, au Nigéria et au Sénégal. Nous analysons comment ces expériences peuvent influencer les droits et l’autonomie des femmes concernant leurs décisions reproductives et leur comportement en matière de contraception. Nous avons utilisé une méthode mixte fondée sur des données issues d’entretiens quantitatifs avec 174 femmes et d’entretiens approfondis qualitatifs avec 10 maris d’un sous-ensemble des femmes dans les trois pays. Les conclusions montrent que les maris ont apprécié les caractéristiques de l’AVP se rapportant à la maîtrise par l’utilisatrice (les femmes pouvaient insérer et retirer elles-mêmes l’anneau), la facilité d’utilisation et la non-interférence avec les rapports sexuels et le flux de lait maternel. La perception par les épouses des expériences de leur mari concernant la non-interférence de l’AVP avec les rapports sexuels cadrait avec les propres affirmations des maris. De plus, les prestataires de soins de santé ont joué un rôle important pour soutenir l’utilisation durable de la méthode en donnant des informations, en conseillant et en aidant les femmes dont l’anneau avait glissé à surmonter ces difficultés. Les conclusions semblent indiquer que des technologies de santé autogérées comme l’AVP pourraient élargir les choix offerts aux femmes et leur maîtrise des décisions reproductives. Elles indiquent également que l’utilisation durable de ces produits pourrait nécessiter des liens avec des structures de santé appropriées pour s’attaquer aux obstacles qui contrarient l’utilisation, au moment et à l’endroit où ils apparaissent.

## Resumen

Con el lanzamiento del Anillo Vaginal de Progesterona (AVP) en África subsahariana se busca entender la función del producto en promover la autonomía de las mujeres en su toma de decisiones y sus comportamientos reproductivos. Ese conocimiento podría informar el posicionamiento del método en los programas de planificación familiar en la región. En este artículo exploramos las experiencias de los esposos con el AVP según las percepciones de sus esposas y los relatos de esposos en un subconjunto de mujeres usuarias en Kenia, Nigeria y Senegal. Discutimos cómo esas experiencias podrían influir en los derechos y en la autonomía de las mujeres en sus decisiones reproductivas y su comportamiento anticonceptivo. Utilizamos el enfoque de métodos mixtos, basándonos en datos de entrevistas cuantitativas con 174 mujeres y entrevistas a profundidad cualitativas con 10 esposos del subconjunto de mujeres en los tres países. Los hallazgos muestran que los esposos valoraron los atributos del AVP relacionados con el control por la usuaria (las mujeres podían introducir y extraer el método por sí mismas), la facilidad de uso y la no interferencia con el sexo ni con el flujo de la leche materna. Las percepciones de las esposas sobre las experiencias de sus esposos relativas a la no interferencia del AVP con el sexo coincidieron con los relatos de los esposos. Además, los prestadores de servicios de salud desempeñaron papeles importantes en apoyar el uso sostenible del método mediante el suministro de información y consejería, y asistiendo a las mujeres que experimentaron deslizamiento del anillo para manejar esos retos. Los hallazgos indican que la autogestión de tecnologías de salud como el AVP podrían ampliar las opciones de las mujeres y su control sobre sus decisiones reproductivas. Sugieren también que el uso sostenible de dichos productos podría necesitar vínculos con las estructuras correspondientes de los sistemas de salud para abordar los retos relativos al uso si y cuando surgen.

## Introduction

The progesterone vaginal ring (PVR) was developed by the Population Council to serve the contraceptive needs of postpartum women since it contains progesterone as the sole hormone as opposed to oestrogen, which is not recommended for lactating women, especially during the first six months after a birth.^1–3^ Studies in multiple settings have shown that PVR is safe and effective, enhances the amenorrhoeic effects of breastfeeding, and is efficacious as long as the mother is breastfeeding at least four times a day.^4–7^ The ring is self-inserted and user-controlled; women can self-insert and remove it when not in need, which gives them greater control over how the product is used.^1,2^ A woman can use up to four rings over a period of 12 months, with each ring lasting three months, before transitioning to another method after one year of use.^1,2,5,6^

Besides expanding contraceptive choices for lactating women, PVR’s attributes might enhance women’s autonomy over their reproductive decision-making and behaviour, and reduce their dependence on husbands as well as health care providers.^1,2^ The potential for PVR to enhance women’s autonomy is because the method is a self-care product that women can use and stop using when they want, reducing dependency on the health system for insertion and/or removal. PVR could further enhance autonomy because the very act of insertion and removal by oneself gives a woman confidence in her ability to control her reproduction. In addition, reduced dependence on health care providers could ultimately reduce the burden on the health care system. The method is also ideal for settings where breastfeeding is near-universal and the availability of skilled family planning service providers is limited, such as in sub-Saharan African (SSA).^1^

While the PVR has potential as a self-care product to enhance women’s autonomy, the effectiveness of self-care interventions for sexual and reproductive health (SRH) may vary by context^8–10^ In the case of PVR, the extent to which it could enhance women’s autonomy over their reproductive decision-making and behaviour may be influenced by cultural acceptance of family planning and vaginally-inserted products, unequal gender and power relations within households, the cost of accessing the product, and ease of use.^11,12^ Studies in SSA further show that socio-cultural norms around gender roles, sexuality, and fertility exert considerable influence on contraceptive use among women and girls in the region.^13–19^

In order to ensure maximum benefits from self-care interventions for SRH, the World Health Organization (WHO) recommends that such interventions encompass strategies that promote active participation of individuals in their health while at the same time enabling them to remain connected to the health care system.^10^ PVR is a user-controlled contraceptive method that is undergoing introduction in selected SSA countries following acceptability studies that were conducted between 2013 and 2014 in Kenya, Nigeria, and Senegal.^2^ At the time of writing this paper, the product had already been registered in Senegal and Nigeria. The introduction of the method in SSA suggests a need for insights on the product’s role in promoting women’s autonomy regarding their reproductive decision-making and behaviour in a context of unequal gender and power relations. Such insights could be important for informing positioning of the method within family planning programmes in these countries, especially decisions related to distribution channels and user information.

In this paper, we examine husbands’ experiences with PVR, first, as perceived by their wives who participated in the acceptability studies conducted in Kenya, Nigeria, and Senegal, and second, as reported by husbands of a subset of the women. The aim was to explore how husbands’ experiences with a self-use product developed for women might influence women’s rights and autonomy regarding their reproductive decisions and behaviour in an environment characterised by gender imbalances around such decisions. We focus on lactating mothers seeking family planning services, who may be at risk of gender-based violence if their actions go against their husbands’ wishes, especially in patriarchal societies such as the study settings where men’s views and perspectives shape women’s choices, decision-making and behaviours.^20,21^ We particularly focus on: (1) husbands’ concerns and experiences with PVR, which could influence their willingness to support their wives using the product; (2) the potential role of PVR in enhancing women’s autonomy in reproductive decision-making and behaviour from the perspectives of their husbands; and (3) the role of the health care system in PVR use from the perspectives of husbands, which could influence women’s agency to use the method. We further draw out implications of such experiences for use of similar self-care health products in other contexts.

## Study setting

Patterns of contraceptive use vary between the three study countries. More than half (58%) of married women aged 15–49 years in Kenya use any method of contraception while 53% use a modern method.^22^ There are, however, wide variations in modern contraceptive use between counties, from 2% in Wajir and Mandera counties to 76% in Kirinyaga county.^22^ The method mix is dominated by injectables, followed by implants and pills.^22^ The overall discontinuation rate for all methods for any reason within 12 months of initiating use is 31%, with the major reasons being side effects/health concerns, desire for a child, becoming pregnant while using, infrequent sex, and desire for a more effective method.^22^ Discontinuation due to husband/partner disapproval and marital dissolution – which could be indicative of unequal gender and power relations – accounts for 2% of the episodes.^22^ More than half (54%) of married women in the country participate in household decisions regarding their own health care, major household purchases, and visits to their family or relatives.^22^

Seventeen percent of married women aged 15–49 years in Nigeria use any method of contraception, with 12% using a modern method.^23^ Use of modern methods ranges from 2% in Yobe state to 29% in Lagos.^23^ Implants and male condoms are the dominant methods, followed by injectables and withdrawal.^23^ The rate of discontinuation for all methods for any reason is higher than for Kenya (41%), while the major reasons for discontinuation include desire for a child, becoming pregnant while using, side effects/health concerns, infrequent sex, and desire for a more effective method.^23^ Four percent of episodes of discontinuation are due to husband/partner disapproval or marital dissolution.^23^ One-third (34%) of married women in the country participate in household decisions regarding their own health care, household purchases, and visits to their family or relatives.^23^

In Senegal, 27% of married women aged 15–49 years use any method of contraception while 26% use a modern method.^24^ Use of modern methods varies from 11% in Matam region to 35% in Saint Louis.^24^ Implant is the dominant method, followed by injectables, pills, and intrauterine device (IUD).^24^ The overall discontinuation rate for all methods for any reason is 29%, and the major reasons for discontinuation include desire for a child and side effects/health concerns.^24^ Discontinuation due to husband/partner disapproval and marital dissolution account for 7% of the episodes.^24^ Ten percent of married women in the country participate in household decisions regarding their own health care, household purchases, and visits to their family or parents.^24^

## Methods

### Data and approach

We use a mixed-methods approach drawing on quantitative data from structured interviews with women who participated in the PVR acceptability studies in the countries, and qualitative interviews with husbands of a subset of the women. The overall acceptability study used a mixed-methods approach, and entailed structured interviews with women who took up the ring, in-depth interviews with a subset of the women, in-depth interviews with the husbands, self-administered semi-structured interviews with health care providers, and focus group discussions with community and religious leaders. In this paper, we specifically used the triangulation design^25^ whereby we drew insights on husbands’ experiences with PVR by supplementing qualitative information from husbands’ reports of their own experiences with their wives’ perceptions of those experiences obtained from the structured interviews. The acceptability studies were conducted mostly in urban and peri-urban settings in all three countries between November 2013 and July 2014. Since PVR was a new method, the choice of the settings was informed by the need to assess its acceptability in settings where contraceptive use was already high. Although PVR is designed for self-use, the acceptability studies were conducted in family planning service delivery points in selected public health facilities in the three countries. The method was provided as part of the method mix at no cost to users, and only women who chose PVR after counselling were recruited into the study.

Women who participated in the structured interviews were those who chose PVR during family planning counselling and who were prospectively followed over a period of six months for two ring cycles. They were interviewed at the time of recruitment and at three and six months of ring use or at the time of discontinuation. Out of a total of 174 women who were recruited in all three countries (60 in Kenya, 58 in Nigeria, and 56 in Senegal), 110 completed the first ring cycle (29 in Kenya, 40 in Nigeria, and 39 in Senegal) while 94 completed the second cycle (25 in Kenya, 38 in Nigeria, and 31 in Senegal). Details about the study procedures, including sample size determination and screening process, are documented elsewhere.^2^ In addition to individual experiences, women were asked about their perceptions of their husbands’/partners’ experiences, including whether the partner felt the ring during sexual intercourse, whether it affected the partner’s sexual pleasure, and for those who discontinued use, whether the partner was responsible for such decisions. Women who discontinued use were those who were successfully tracked and who were determined to have stopped using the method while those who could not be traced were considered as lost to follow up. The interviews with women who returned after each ring cycle took place at the health facilities while those who discontinued and did not return but were successfully traced were interviewed in their homes.

In-depth interviews with husbands of PVR users were conducted at the end of the two ring cycles. In order to minimise the risk of partner violence against those who could be covertly using the method, women were first asked whether their partners would be willing to participate in an interview. Only husbands of those women who were agreeable to their partners being interviewed were approached for this purpose. The husbands were approached and interviewed in their homes after the women linked the interviewers to them. A total of three husbands were targeted in each country based on available resources for conducting the overall acceptability study. All husbands whose partners were agreeable to them being interviewed accepted. Participants were asked about decision-making regarding PVR use, initial impressions about the ring, their experiences and the experiences of their partners with the ring, sharing of such experiences with friends, and opinions regarding the future of the ring (see Appendix 1 for the list of questions). Interviews were conducted by trained personnel in the dominant national languages spoken in the countries – Kiswahili in Kenya, English and/or Pidgin English in Nigeria, and Ouolof in Senegal. Interviews in Kenya and Senegal were conducted by male study coordinators, and in Nigeria, by a male research assistant with extensive experience in qualitative data collection. The use of male interviewers fostered openness on the part of the participants, especially with respect to giving information about sensitive issues such as sexual experiences during PVR use. The interviews were audio-recorded with written consent of participants and transcribed in French and English and the French transcripts were then translated into English. There was no back-translation of the transcripts to determine if any meanings were lost in the process.

### Analysis

Analysis of the quantitative data entailed generating descriptive statistics (percentages) to determine the extent of partner experiences with the ring as perceived by their wives. Analysis sample was the number of cycles of ring use given that wives were asked about their perceptions of their partners’ experiences at each cycle. Thus, a woman who completed only one cycle or discontinued during the first cycle contributed one episode to the analysis while one who completed two cycles or discontinued during the second cycle contributed two episodes. Analysis included a total of 223 episodes. Decisions regarding discontinuation were, however, asked only once and included 34 women who discontinued the method during the first or second cycle. Quantitative data analysis was conducted using Stata® 16 software.

We used an exploratory inductive content analysis approach to analyse the qualitative data from interviews with men. This approach was taken because we had no preconceived notions of what the data would reveal. All authors read and re-read the transcripts and outlined emerging themes, supported by excerpts from the transcripts, based on their understanding of the husbands’ narratives in relation to the potential for PVR to influence women’s autonomy regarding reproductive decisions and behaviour. The themes were then compared across authors and any discrepancies were resolved through a series of discussions that were held virtually. We use excerpts from the transcripts to illustrate aspects of men’s experiences with PVR that could have implications for women’s autonomy, and the place of the health care system in the use of this self-care technology.

### Ethical considerations

The Population Council’s Institutional Review Board (Protocol 562 approved on 6 November 2012) and relevant research and ethics bodies in Kenya, Nigeria, and Senegal granted ethical approval for the study. In Kenya, approval was granted by the Kenyatta National Hospital/University of Nairobi Ethics and Research Committee (Protocol number P625/11/2012 approved on 13 February 2013) and the National Council for Science, Technology, and Innovation (Reference number NCST/5/002/R/683 granted on 13 March 2013). Ethical approval in Nigeria was granted by the Federal Capital Territory (FCT) Health Research Ethics Committee (Protocol number FHREC/2012/12/39/13-12-12 approved on 12 December 2012) and the Institute for Advanced Medical Research and Training (IAMRT) (Protocol number UI/EC/12/0376 approved on 15 November 2012). In Senegal, the study received approval from the Comité National d’Ethique pour la Recherche en Santé (CNERS) (Protocol number SEN12/71 approved on 13 February 2013) and the Department of Planning Research and Statistics (Protocol number SEN12/71 approved on 13 February 2013). All participants provided written informed consent before being interviewed, with consent from women being obtained at each round of interview.

## Results

### Characteristics of participants

Ten husbands across the three countries (three in Kenya, three in Nigeria, and four in Senegal) participated in the in-depth interviews. In all cases, their partners completed two ring cycles. The interviews lasted between 30 and 60 minutes. The study did not capture the demographic information of husbands who participated in the interviews. Demographic information of women who participated in the quantitative interviews was captured at the time of recruitment into the study. The median age of the women was 28 years (range: 18–35 years). Sixty percent of the women had secondary or higher levels of education while only 1% was not in a marital relationship (not married or cohabiting) at the time of recruitment. The majority of the women were from either urban (45%) or peri-urban (43%) settings. The results on husbands’ experiences with PVR are organised by major themes from the qualitative interviews. We use insights from quantitative interviews with the women to support some of the themes from the qualitative interviews.

### Husbands’ initial concerns about PVR

Qualitative interviews with husbands showed that their initial concerns with PVR revolved around fear of a new product, the effect it could have on their partners’ health, whether it could disrupt sexual intercourse or sexual lives in general, and whether it was effective at preventing pregnancy.

#### Concerns about PVR’s relatively big size and shiny texture

The narratives showed that some husbands were initially concerned about PVR’s size and texture when they first saw the method. Some participants reported that they initially thought the ring was hard and wondered how it could fit into the vagina or whether it could slip into the womb. As one husband from Nigeria narrated, “*Wow!! (Laughter) I thought it was metal! … I was thinking how can she be walking around with metal … Won’t this thing fall out? Can she walk well with it? … You know, will it be painful?*” (Husband #3, Nigeria). Two participants from Senegal were also initially concerned about the size of the ring and whether it could fit into the vagina. As one participant put it, “*Initially I had apprehensions about size and asked about how could one use such a product,*” (Husband #2, Senegal). For some, such initial impressions generated fear around a new product, as narrated by a participant from Kenya: “*When I saw it, I thought it was replica of the coil. I was even convinced that it was the coil since I didn’t see the difference between the two … I was so scared, having heard a lot of rumours about family planning*” (Husband #3, Kenya).

#### Concerns about potential known and unknown side effects of PVR

Other participants were concerned about the ring having unpleasant side effects that would affect their partners’ health. Some participants explicitly referred to concerns about known side effects of contraception such as excessive bleeding. A participant from Kenya, for instance, thought that the ring “* … may cause excessive bleeding or cramps”* or “*affect breast milk*” (Husband #3, Kenya). Other participants referred to general challenges such as “*consequences on a woman’s health*” (Husband #1, Senegal), and “*interference with the body*” (Husband #3, Senegal), without specifying what those consequences or interferences were. Another participant from Kenya was concerned about the method causing cancer, which is not a medically proven side effect of contraception. The participant stated that, “*Nowadays, we have chronic diseases like cancer, so I needed to know if it had such adverse effects*” (Husband #2, Kenya).

#### Concerns about potential interference with sex

Husbands across the three settings were initially concerned about PVR’s interference with sex. There were concerns that the ring would make sex painful for both the wives and their husbands. As a participant from Nigeria narrated, “*On my own side I was thinking, ‘Won’t this thing wound me? (laughter) Wow … Yes, I thought … of her having pains during intercourse*’” (Husband #3, Nigeria). Another participant from Senegal reported that, “*When I saw the ring for the first time, the first question that touched me is ‘is the ring not going to bother me during sex’ … that it was too big and I wondered if it could interfere with sex*” (Husband #4, Senegal). Similar sentiments were expressed by a participant from Kenya, “*it might hurt during sex*” (Husband #3, Kenya).

#### Concerns about potential ineffectiveness of PVR

Some participants were concerned about the effectiveness of PVR at preventing pregnancy. One participant from Kenya alluded to not understanding the science of how the ring works to prevent pregnancy and that the decision to use the method was a gamble:

“*I didn’t understand the science of how it prevents pregnancy … I did not trust the gadget at first, so I was worried she might get pregnant. I told you it was like gambling. I didn’t understand the science but decided to go use it.*” (Husband #2, Kenya)

Another participant from Nigeria wondered how the method works and whether his wife would not become pregnant again, “* … and also will this thing work? Won’t she get pregnant again?*” (Husband #3, Nigeria).

#### Husbands’ positive and challenging experiences with PVR

Husbands’ reports of their own and their partners’ experiences with PVR allayed the concerns they initially had about the method. Their experiences with PVR were largely positive and included ease of use and user-control, absence of side effects, non-interference with sexual intercourse, and effectiveness of the method at preventing pregnancy. The major challenge that some participants reported was related to ring slippage.

#### Ease of use and opportunity for user control

Despite initial concerns about the size and texture of the ring, husbands across the three settings reported that the ring was easy to use. Most participants referred to the method as being “user-friendly”. As one participant from Senegal reported,

“*My wife didn’t have any problems using the method which was easy to use. She didn’t complain much … I had no worries and the fact that it was in my wife’s body (vagina) did not cause me any problems … The use of the ring is very easy if I refer to the experience and explanations of my wife.*” (Husband #1, Senegal)

Similar sentiments were expressed by another participant from Senegal:

“*What I liked about the ring is its ease of use. Also an illiterate and an educated woman can use it easily … My wife throughout the period had no difficulty using the ring. It was easy to use … my wife had not had a problem during the whole time she was using the ring.*” (Husband #3, Senegal)

Some participants appreciated the fact that their wives had control over the use of the method. A participant from Nigeria reported thus, “*Yes, I like it … when you want, you can take it out if you don’t want to use it … I think it is easy. Because sometimes, I asked her whether it was disturbing her and she said no”* (Husband #1, Nigeria). Another participant from Senegal reported that PVR is “*easy to use, free, self-controlled method by the woman herself”* (Husband #4, Senegal).

#### Absence of side effects

Participants reported absence of side effects and less inconvenience with the method compared to other methods, including non-interference with the flow of breast milk and their partner’s weight. Absence of side effects was largely expressed in terms of the wife having no problems or complaints when using the ring. Two participants from Kenya reported that the method did not “*interfere with [production of] breast milk”* (Husband #2 and #3, Kenya). Another participant from Senegal reported that “*I appreciate … the absence of side effects … My wife has never had a problem with its use*” (Husband #2, Senegal).

#### Non-interference with sexual intercourse

Some participants reported not feeling the ring during sexual intercourse, and that there was no change in their experiences during sex. Those who indicated that they felt the ring reported that it did not interfere with sex or that it resulted in pleasurable feeling during sex, which some participants appreciated. A participant from Kenya reported that,

“*There was also the sucking effect. I did not know if it was just from my end, but I could feel it from the ring itself. If you feel it, of course you happen to penetrate in between the ring, you feel like something is sucking. It is a good feeling*.” (Husband #2, Kenya)

Another participant from Senegal reported that, “*The ring never bothered me during sexual intercourse. The ring has not changed our habits in our sexual relations in terms of frequency or sexual pleasure. For me, the situation was the same compared to the past*” (Husband #2, Senegal).

Wives’ perceptions of their husbands’ experiences with the ring during sexual intercourse were consistent with the husbands’ reports for the most part. Wives reported in 65% of the cases that their husbands never felt the ring during sexual intercourse (Table 1). They further reported in about three-quarters of the cases that the ring never affected their husbands’ sexual pleasure or that there was no change in their husbands’ sexual pleasure (76% and 74% of the cases, respectively). Changes in husbands’ sexual pleasure were also more positive than negative, with a higher proportion of cases involving an increase than a decrease in pleasure (18% and 4%, respectively; Table 1). The patterns were consistent with women’s reports of their own experiences with PVR during sexual intercourse. Women reported that they did not feel the ring during sex in 73% of the cases, that the ring never affected their sexual pleasure in 80% of the cases, and that there was no change in the frequency with which they had sex in 75% of the cases. Where women reported changes in frequency of sex, it was more of an increase than a decrease in such frequency (19% and 4% of the cases, respectively).

**Table 1.** Women’s perspectives of husbands’ experiences with PVR in Kenya, Nigeria and Senegal

Indicator	Percent
Husband felt the ring during sexual intercourse	(*N* = 223)^a^
No, never	64.6
Yes, sometimes/always	29.6
Don’t know/did not answer	5.8
Ring affected husband’s sexual pleasure	(*N* = 223)^a^
No, never	76.2
Yes, sometimes/always	15.3
Don’t know/did not answer	8.5
Change in husband’s sexual pleasure when using the ring	(*N* = 223)^a^
No change	74.0
Increase	17.5
Decrease	3.6
Don’t know/did not answer	4.9

^a^ Total number of cycles of use among women interviewed at 3-month and/or 6-month follow-up in all three countries.

#### Effectiveness of PVR at preventing pregnancy

Other participants reported that the method was effective because their partners did not experience unintended pregnancy when using the method. A participant from Kenya reported that he did not have any issues with the method because it “*was very effective for the six months that she [his wife] used it*” (Husband #2, Kenya).

#### Challenges with ring slippage

Despite the positive experiences, a few husbands reported some challenges when using the ring. One participant from Kenya and two from Senegal reported that their partners experienced slippage of the ring. One participant from Nigeria reported experiencing unpleasant odour after two to three months of his partner using the ring during the two cycles. Three participants (one from each of the three countries) reported initial discomfort with the ring during sexual intercourse partly due to feeling the ring and partly due to psychological awareness of its presence in their partners’ body. A participant from Kenya reported, thus,

“*At times, you don’t realise it is there, but it will remain in your mind that it is there, and of course you will feel it … At first, I told you it was not comfortable but it had a lot to do with the mind set because I knew there was something inside there, so I did not trust it at first. So, it was as a bit tricky. I could feel it, but like I said, the feeling was not painful or anything, just something soft*.” (Husband #2, Kenya)

Similar sentiments were expressed by a participant from Senegal:

“*Sometimes the ring was slipping, though it was not common. We were aware that with the presence of the ring, our sexual relations would be different. We knew there was a foreign body and it had a little psychological effect but it did not bother us at all.*” (Husband #3, Senegal)

Such challenges did not, however, affect their attitudes towards the method or their relationships with their partners as shown by the following quote from Nigeria, “*Well, it was not like before, but there is nothing I could do … I explained to her … I explained saying that this place, it was not like before … We have to manage it like that*” (Husband #1, Nigeria).

### PVR and women’s reproductive autonomy

We explored the role of PVR in women’s autonomy in the context of decision-making regarding contraception and choice of the method, women’s ability to self-use the method and manage associated challenges such as ring slippage, and husbands’ role in discontinuation of the method and contraceptive switching after two ring cycles.

#### Joint or consultative decision-making regarding contraception

Qualitative interviews with husbands showed that the choice of PVR was preceded by deliberate decisions by the couple to use a family planning method to prevent an unintended pregnancy. The decision to use a method in some cases came from the husband while in other cases it was made jointly. As one participant from Senegal reported, “*My wife and I had discussed the need to space births … Our wish was a spacing between 2 to 3 years*” (Husband #1, Senegal)*.* Another participant from Nigeria reported that “*That time I said, ‘Let us find the solution’ [to spacing births] … She said okay. I let her go to hospital*” (Husband #1, Nigeria).

The decision to use PVR was also either made jointly in cases where husbands accompanied their partners to the facility, or husbands were consulted before or informed after their wives obtained the method in cases where the women were not accompanied by their partners. Husband #1 from Senegal mentioned above reported that, “*After receiving more detailed explanations of this method, she finally opted for the ring. I was not informed until after her return*”*.* Similar sentiments were expressed by Husband #1 from Nigeria,

“*She did not discuss [PVR] with me but after she saw it … she said, ‘Let me try it. If the thing works, I will continue’ … I responded, ‘Good, because it is when you try it that you can see whether it is good.*” (Husband #1, Nigeria)

Another participant from Kenya reported,

“*I was not involved at this particular stage, but I had suggested to her that I wanted her to start using family planning. So, when she was with the nurse, she knew this is what I wanted and when she came out [of consultation], she had been served with it [PVR].*” (Husband #1, Kenya)

The narratives further showed that the choice of PVR was informed by the desire for a more effective method with no side effects, its being free and curiosity around a new product.

#### Ability to manage self-use and challenges associated with use

As previously indicated, most husbands reported that PVR was easy for their partners to use, and that their partners had control over when to insert or remove it. A majority of the women (more than 80%) also found the ring easy to insert, remove, and re-insert. However, two husbands (one in Kenya and the other in Senegal) reported that their partners experienced challenges when the ring slipped and that they had to seek help from the health facilities where they obtained the method.

#### Minimal role of husbands in discontinuation and switching

**Figure 1.**
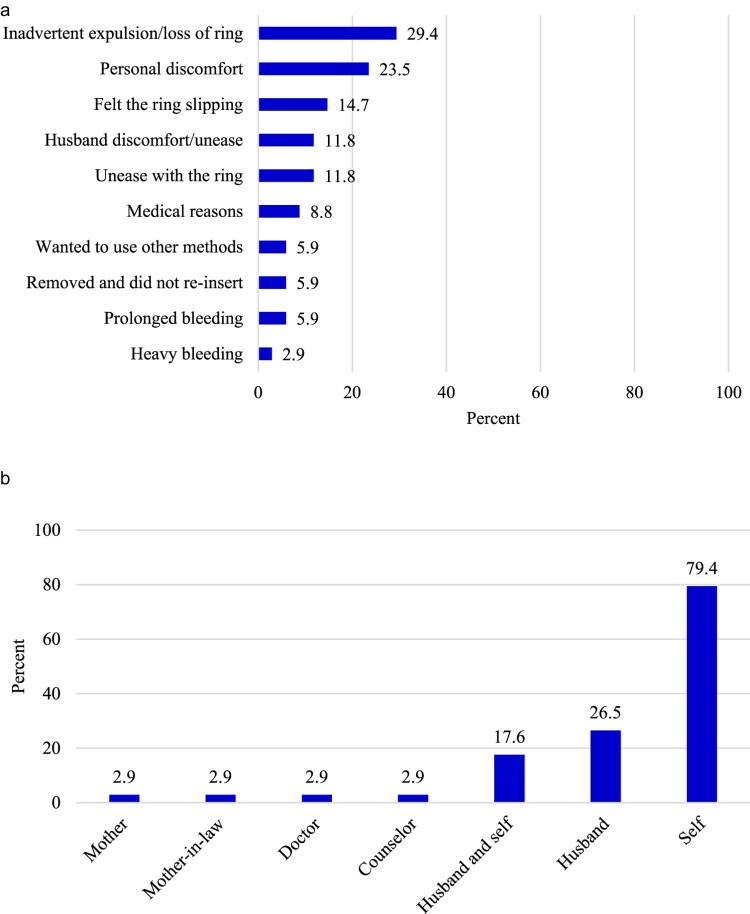
a: Reasons for PVR discontinuation among women in Kenya, Nigeria and Senegal b: Decision-maker regarding PVR discontinuation Note: Multiple responses were allowed in both cases; Number of discontinuations: 34; Source: Women’s interviews

Interviews with women who discontinued using PVR showed that their husbands played a minimal role in such decisions. Twelve percent of the women who discontinued using the method did so due to husband discomfort or unease with the ring (Figure 1(a)). However, the most common reasons for discontinuation pertained to the individual woman’s experiences with PVR, including inadvertent expulsion or loss of the ring, personal discomfort, and feeling the ring slipping (Figure 1(a)). In addition, the decision to discontinue using the ring was mostly made by the wife (79% of the cases) while husbands made such decisions in 27% of the cases (Figure 1(b)).

Most husbands reported that their partners switched to other methods (injectables, pills, implant, IUD, or condoms) after two ring cycles and only two (one in Kenya and one in Senegal) did not know the method while one (in Nigeria) reported that his partner had not taken up a method. A participant from Kenya, for instance, reported that “*I don’t know. I didn’t ask her if the one she is using is any different from the ring … I didn’t have time to come with her to the hospital, but she told me she was given a method*” (Husband #1, Kenya). Another participant from Nigeria narrated that, “*Apart from that tablet you people gave her, I don’t think there is anything else … She just came back with it. When I asked her about it, she said it is the substitute method you people gave her*” (Husband #3, Nigeria). From Senegal, a participant reported that,

“*I don’t know exactly, but I think it’s a method she places in her vagina [IUD] … I was not involved in the choice of this new method but I agree with this choice as long as there are no side effects.*” (Husband #4, Senegal)

However, all participants reported that wives should inform their husbands before using PVR or any other family planning method in order to avoid marital conflicts.

### PVR and the importance of the health system

The narratives from husbands showed that health care providers played an important role in giving information about the method; showing women who chose it how to insert, remove, and re-insert; and counselling them on potential side effects and what to do in case of ring slippage or expulsion. Some husbands who accompanied their partners to the health facilities reported being informed about the method by health care providers during consultations. A participant from Kenya, for instance, reported that

“*We were visiting the clinic, then a nurse introduced it to us. She explained to us how it works and what it looks like, and also told us that it was a new product … After talking with them, we went somewhere to discuss about it and we came up with the final decision*.” (Husband #3, Kenya)

In some cases where the husband did not accompany the wife to the health facility, he was informed of the role health care providers played in giving information and showing women how to use the method as exemplified by the description of one participant from Senegal, thus: “*She could insert it and remove it without difficulty because she had told me that the midwife had shown her how to do it*” (Husband #4, Senegal).

The role of health care providers was further evident from other settings as reported by a participant from Nigeria,

“*That day in the hospital, they showed her different methods; they explained it to her. She said, okay, let her try that ring. So, that is how we came to a conclusion … I was there … When they were explaining everything, I saw it in the almanac [banner]. So, when they were talking about it, I was reading about it by myself*.” (Husband #3, Nigeria)

A husband from Senegal also reported participating in the counselling session:

“*I was the one who accompanied her to the health center and I even participated in the counselling session. I can say that I even influenced the choice because the presentation of the method was clear and for me this method presented less disadvantages and risk compared to others … After counselling, we decided together to take this new method*.” (Husband #3, Senegal)

It was evident from some of the narratives from husbands that despite receiving initial information and counselling about the method from health care providers, some couples still had challenges re-inserting the ring whenever it came out and had to seek help from the health facilities where they obtained the method. The narratives showed that health care providers played an important role in supporting couples who had challenges with re-insertion after experiencing ring slippage. As one participant from Senegal reported,

“*Initially, my wife had some problems because the ring often slipped. She told me about it and went back to the [health] center and was told what to do. And from then, she managed to insert the ring herself if it came out*.” (Husband #1, Senegal)

Another participant from Kenya reported unsuccessfully trying several times to re-insert the ring for her partner until they had to go back to the health facility for help, thus:

“*There is only one problem with the PVR: It fell out a couple of times and we were not able to return it correctly. It fell out even when she visited the toilet, and it was quite unhygienic to reinsert it … It forced us to go back to the hospital again for it to be inserted once more … So many times I tried myself to insert it but was unable to place it in the right way and it would come out after two days again*.” (Husband #3, Kenya)

However, some participants appreciated the fact that PVR did not require frequent facility visits, thereby reducing the cost (both in terms of time and money) of seeking care.

## Discussion and implications

Self-care interventions for SRH provide opportunities for achieving positive health and social outcomes both for service users and the health care system, especially in settings experiencing challenges with human resources for health or in situations where formal interaction with health care providers is restricted, such as during the global COVID-19 pandemic.^10,26–28^ Such outcomes include improved access to services in terms of wider coverage and reduced costs, promotion of equity and user autonomy, and reduced burden on the health care system^10,26,27^ In the context of PVR, the findings of this study show that user control, ease of use, and non-interference with sex and flow of breast milk were attributes of the method that husbands appreciated. This was consistent with previous findings regarding the views of women about the method.^2,12^ Such positive views might contribute to men’s support for their partners to use the method, and thus improve the SRH and rights of women in the study settings. The finding further suggests that husbands could play an important role in promoting PVR if they find the method acceptable, and thus contribute to expanding the SRH and rights of women generally.

All husbands interviewed across the three study settings considered it important for their partners to inform them when using PVR or other family planning methods to avoid conflict within households. Whereas this view may negate the goal of achieving SRH and rights for women through self-use products such as PVR, a previous study showed that women were also of the view that they needed to inform their partners to ensure harmony in the home.^12^ In patriarchal societies such as the study settings, men’s views and perspectives often shape the choices available to women, women’s decision-making, women’s own formation of opinions, and women’s behaviours.^20,21^ The social position of men in the study communities suggests that their support is crucial for enhancing women’s sustainable use of PVR given the considerable influence they have on the SRH and rights of women. The finding suggests that as PVR undergoes registration in SSA countries, there is a need for strategies for engaging men while ensuring that the reproductive autonomy (user-control) that the ring offers women is not compromised.

One strategy that worked for PVR in the study context was the involvement of husbands in decision-making and counselling about the method. Husbands who were involved in decision-making or counselling sessions supported their partners to use the method, including managing challenges associated with ring slippage. The importance of partner support is consistent with WHO’s recommendation that self-care interventions should be implemented in an environment that considers individual circumstances and the conditions under which people live.^10^ However, as with other methods, PVR may not be suitable for every couple as some husbands may not support their wives’ use of the method. If the wife prefers the method but the husband does not, programmes have to devise alternative ways of counselling the user. In such cases, women should be counselled alone, told what the male partner may know or feel if she is using the ring, or provided with information and support that address her need for confidentiality. Ultimately, the decision to use PVR or not, and to be counselled separately from husband or not, should be made by the wife. Women who want autonomy over their reproductive choices and who wish to use PVR covertly due to opposition from their partners should be supported in their choices. A woman who wants to use PVR covertly should be informed that some men can feel the ring so that she decides if she wants to keep it during sex or not. PVR and other discreet, self-use products have the potential to enhance women’s autonomy regarding reproductive health in settings where gender relations are unequal or not conducive to women’s own desires about reproduction.

The findings of this study further show that although PVR is a self-use product, health care providers played important roles in supporting its sustainable use through providing the method, giving information, counselling, and assisting women who experienced ring slippage to manage those challenges. This was consistent with findings from a previous study that found that challenges associated with ring slippage and expulsion sometimes required assistance from health care providers.^11^ These insights are consistent with WHO’s recommendation that self-care interventions should not alienate users from the health care system.^10^ Although participants in the present study alluded to ease of using PVR, appropriate use of the method and other self-care interventions for SRH is dependent on the beneficiaries’ ability to understand and adhere to user instructions.^29^ This implies that in contexts of high levels of illiteracy, ensuring appropriate use of self-care interventions requires engagement with individuals and institutions with appropriate expertise to guide users and prevent the risk of such interventions perpetuating rather than reducing inequality in access to care. The registration of PVR in SSA countries therefore suggests a need for strengthening the health care systems in these settings to adequately support the use of such self-care interventions. Once knowledge of the method is commonplace in a community, support and guidance from facility-based family planning providers can decrease. Other mechanisms of support, such as through community-based outreach workers/volunteers or through digital platforms, can emerge. Furthermore, as both users and health care providers become more comfortable with PVR, providers can inform those users who feel the ring during sex that they can remove it but need to re-insert it immediately thereafter. However, providers and users need to balance the benefits of ring removal during sex with the risk of forgetting to re-insert it thereby reducing its efficacy.

Another finding of the study was that nearly all husbands interviewed, except one, reported that their partners switched to another method after completing two cycles of PVR although some participants did not know the method their partners had switched to. Others reported that they were not involved in the choice of the method their partners switched to but supported those choices if they had no side effects. These findings could indicate women’s reproductive decision-making autonomy in switching to other methods after using PVR, especially for those whose husbands were involved in decisions to choose PVR but were not involved in the choice of the methods they switched to. The choice to switch to a new method without informing the partner was also possibly due to prior deliberate decisions by couples to prevent unintended pregnancy. Enabling individuals and couples to freely decide whether or not to use contraception, which methods to use, and whether, when and how many children to have, is at the core of rights-based family planning and the global development agenda.^30–32^ However, poverty, health systems challenges, and retrogressive socio-cultural norms may impede the realisation of the goals of rights-based SRH in resource-constrained settings such as SSA. Self-care health interventions that promote concordance among couples, such as PVR, may therefore contribute to the realisation of such goals in these contexts.

## Limitations

The findings of this study may be influenced by certain limitations. First, data were collected as part of an acceptability study. Although the study mimicked the standard service delivery conditions in the three countries, there were additional procedures involving screening of women who chose PVR during counselling for eligibility to participate in the research. Thus, the wives through whom husbands were identified were a select group that may not be representative of all postpartum women seeking family planning services. Second, since only husbands of consenting wives were interviewed, the findings could be biased towards more favourable experiences of PVR. For instance, the study does not include women who may have husbands who do not support the use of contraceptives generally or those whose husbands oppose the use of hormonal or vaginal methods in particular, and who may have less favourable views about PVR or self-use products. The approach was, however, justified by the need to minimise the risk of partner violence for women who could be secretly using the method. Third, the fact that participants were mostly drawn from urban and peri-urban settings in the three countries, where contraceptive use is high, implies that their views and experiences may not reflect those of rural residents or settings where contraceptive use is low. In addition, even within the urban and peri-urban settings of the three countries, the small sample size of men limits the ability to generalise their views and experiences to all men in such settings. Fourth, husbands’ opinions of and experiences with PVR pertain to the period when the research was conducted and may not reflect the status at the time of writing this paper given that opinions and experiences may change over time.

## Conclusion

In spite of the limitations, the findings of this study highlight the potential for self-use health care interventions such as PVR to expand women’s choices and control over their reproductive decisions. The findings further suggest that sustainable use of self-care health interventions in contexts such as those of sub-Saharan Africa might require linkages with appropriate health systems structures to address challenges with use if and when they arise. In addition, consistent with global recommendations, the findings suggest a need for self-care health interventions to take into account the circumstances in which beneficiaries live to ensure that the interventions expand access to high quality care at affordable costs, rather than perpetuate inequities, while safeguarding the rights of users.

## Implications for policy and practice

- Self-care products such as PVR provide opportunities for enhancing women’s SRH and rights in resource-constrained settings through expanding choice and decision-making regarding their own health care.
- To ensure successful adoption of vaginally-inserted self-care products such as PVR by women in resource-constrained settings, programmes might need to take into account couple relationships in order to safeguard the rights of women.
- Sustainable use of self-care interventions for SRH in resource-constrained settings might require programmes to create linkages with appropriate health systems structures to address challenges with use if and when they arise.

## Data Availability

The qualitative data that support the findings of this study are available on request from the corresponding author [FO]. The data are not publicly available due to their containing information that could compromise the privacy of research participants. The quantitative datasets that do not contain information that can be used to identify participants are available at: https://dataverse.harvard.edu/dataset.xhtml?persistentId=doi:10.7910/DVN/RYLSTW.

## Appendices

### Appendix 1: Qualitative interview questions with husbands

1. Let’s start with your initial perception of the PVR. Your wife has successfully used the vaginal ring for a six-month period. In general, what do you think about the vaginal ring as a method of family planning? What did you think when you first came across the vaginal ring? What was your reaction?
2. What exactly did you like about the ring? Compared to the other methods, what do you like about this ring? What didn’t you like or what did you like least about the ring?
3. Describe how your wife or partner ended up choosing the vaginal ring as a family planning method. To what extent were you involved in choosing this method?
4. Now let’s look at your experience with the ring. How easy do you think it was for your wife to use the vaginal ring? What effects did the ring have on your sex life, or on that of your partner while she was using this ring?
5. Do you think women using the ring should inform their sexual partners? Please, explain the reason for your response.
6. Now, let’s talk about sharing experiences with friends. Now that your wife has had a chance to use the vaginal ring, would you encourage your friends to have their partners try the ring? Please, explain the reason for your response.
7. Do you think the vaginal ring should be provided as a regular method of family planning in this country? Please, explain the reason for your response.
8. After experiencing the ring through your wife, what improvement would you like introduced to this ring?
9. The ring your partner used was only meant for 6 months and was for lactating mothers only. How would you feel about your wife using a ring that is independent of breastfeeding and that can be used for one year, and is removable every 3 months?
10. Would you mind if your wife used a multi-purpose ring that protects her from STIs like HIV, and against pregnancy? Please, explain the reason for your response.
11. Now that your wife has reached the end of the 6-month period, what method of family planning is she using and how did she choose the method?
12. We have come to the end. Do you have anything you would wish to add?

### Appendix 2: Structured interview questions with women on discontinuation and husbands’ experiences with PVR

**Table:**

DISCONTINUATION QUESTIONS				
1	For how long did you use the Ring before stopping? **[FOR TERMINATION DURING SECOND CYCLE, REFER TO SECOND RING]**	Less than 1 month	1	
		1–2 months	2	
		2–3 months	3	
		Don’t remember	4	
2	What were the reasons for stopping to use the Ring? **[DO NOT READ LIST. CIRCLE ALL MENTIONED AND PROBE BY ASKING ‘ANY OTHER?’]**	Personal discomfort	1	
		Unease with ring	2	
		Removed and didn’t reinsert	3	
		Inadvertent expulsion and loss of Ring	4	
		Feeling the ring slip	5	
		Amenorrhea	6	
		Irregular bleeding	7	
		Prolonged bleeding (>7 days)	8	
		Heavy bleeding	9	
		Medical reasons	10	
		Planning pregnancy	11	
		Want to use other family planning methods	12	
		Reduced breastfeeding (less than 4 breastfeeds per day)	13	
		Husband/partner discomfort/unease with the Ring	14	
		Became pregnant	15	
		Other (specify)_______________________________	16	
		Did not answer	17	
3	Who made the decision that you stop using the Ring? **[DO NOT READ LIST. CIRCLE ALL MENTIONED AND PROBE BY ASKING ‘ANY OTHER?’]**	Self	1	
		Husband/partner	2	
		Mother	3	
		Mother-in-law	4	
		Other family members	5	
		Doctor	6	
		Counselor	7	
		Social worker	8	
		Friends/neighbors	9	
		Other (specify)______________________________	10	
		Did not answer	11	
**QUESTIONS ON HUSBANDS’ SEXUAL EXPERIENCES**				
1	Have you been having sex with your husband/partner SINCE you started using the Ring?	Yes	1	** **
		No	2	**SKIP TO NEXT SECTION**
		Does not remember	3	** **
		Did not answer	4	** **
2	Does your husband/partner always, sometimes or never feel the Ring during sexual intercourse?**[READ OUT LIST. CIRCLE ONLY ONE RESPONSE]**	Yes, always	1	** **
		Yes, sometimes	2	** **
		No, never***]***	3	** **
		Don’t know	4	** **
		Did not answer	5	** **
3	Do you think that the Ring always, sometimes or never affects your husband’s/partner’s sexual pleasure? **[READ OUT LIST. CIRCLE ONLY ONE RESPONSE]**	Yes, always	1	** **
		Yes, sometimes	2	** **
		No, never	3	** **
		Don’t know	4	** **
		Did not answer	5	** **
4	Do you think that there has been an increase, decrease or no change in your husband’s/partner’s sexual pleasure since you started using the Ring? **[READ OUT LIST. CIRCLE ONLY ONE RESPONSE]**	Increase	1	** **
		Decrease	2	** **
		No change	3	** **
		Don’t know	4	** **
		Did not answer	5	** **
